# Supplementary material for: A NIN-LIKE PROTEIN mediates nitrate-induced control of root nodule symbiosis in Lotus japonicus
Source: Nat Commun. 2018 Feb 5;9:499. doi: 10.1038/s41467-018-02831-x (PMC5799372; doi:10.1038/s41467-018-02831-x)
Supplement: Supplementary file 1 — Supplementary Information [file 41467_2018_2831_MOESM1_ESM.pdf]

Supplementary Information

A *NIN-LIKE PROTEIN* mediates nitrate-induced control of root nodule symbiosis in *Lotus japonicus*

Nishida et al.

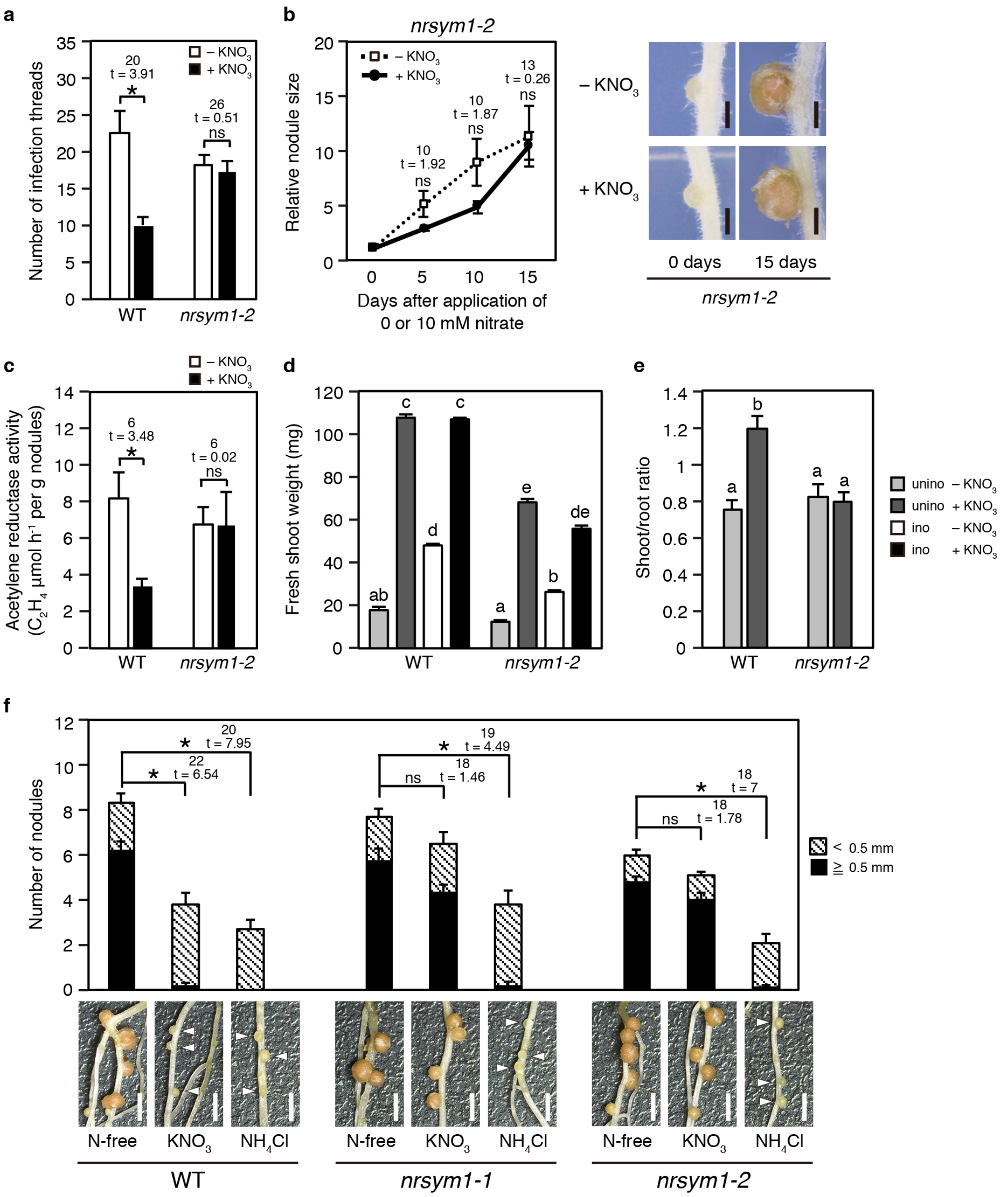

Supplementary Figure 1. Nodulation and plant growth phenotype of the *nrsym1-2* mutant and effect of ammonium on nodulation.

(a) The number of infection threads in WT and the *nrsym1-2* mutants with 0 or 10 mM KNO_3 at 7 dai with rhizobia that constitutively express *DsRED* (n = 14–16 plants). (b) Relative nodule size (daily nodule size/nodule size at day 0) of the *nrsym1-2* mutants (n = 10–18 nodules). Individual nodule size was measured at 0, 5, 10 and 15 days after the transfer to agar plates with 0 or 10 mM KNO_3. **P* < 0.05 (Student's *t*-test compared 0 mM KNO_3-treated nodules with 10 mM KNO_3-treated nodules on the same day). ns, not significant. Scale bars: 0.5 mm. (c) Acetylene reduction activity (ARA; μmol h^{-1} per g nodules) of nodules formed on WT and the *nrsym1-2* mutants (n = 4 plants). Twenty-one dai plants without KNO_3 were supplied with 0 or 10 mM KNO_3, and after 3 days the ARA of nodules from each plant was measured. (d) Fresh shoot weight and (e) shoot to root fresh weight ratio of WT and the *nrsym1-2* mutants grown in 0 or 10 mM KNO_3 at 21 dai (ino) or without rhizobia (union; n = 10–12 plants). (f) Nodulation and nodule numbers of WT, the *nrsym1-1* mutant and the *nrsym1-2* mutant treated with 0, 10 mM KNO_3 or 10 mM NH_4Cl at 21 dai (n = 10–12 plants). Arrowheads indicate small and premature nodules. Scale bars: 2 mm. Error bars indicate SEM. **P* < 0.05 by Student's *t*-test. ns, not significant. Degrees of freedom are shown above the *t*-values (a-c,f). Columns with the same lower-case letter indicate no significant difference (Tukey's test, *P* < 0.05; d,e).

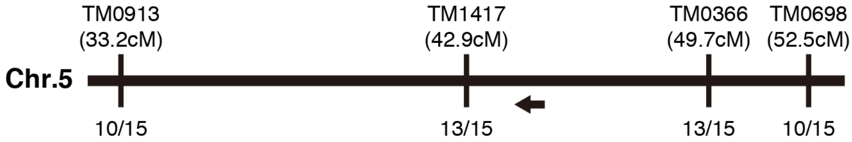

Supplementary Figure 2. Map–based cloning of *NRSYM1*.

The *nrsym1* locus was mapped using an F_2 population derived from a cross between *nrsym1-1* and Gifu B-129 plants. Fifteen F_2 plants that exhibited the nitrate-tolerant phenotype were used for this analysis. The arrow indicates the relative location of the *NRSYM1* candidate gene (chr5.CM0148.170.r2.a (*LjNLP4*)) found in the *L. japonicus* genomic sequence database. The primers used for PCR are listed in Supplementary Table 3.

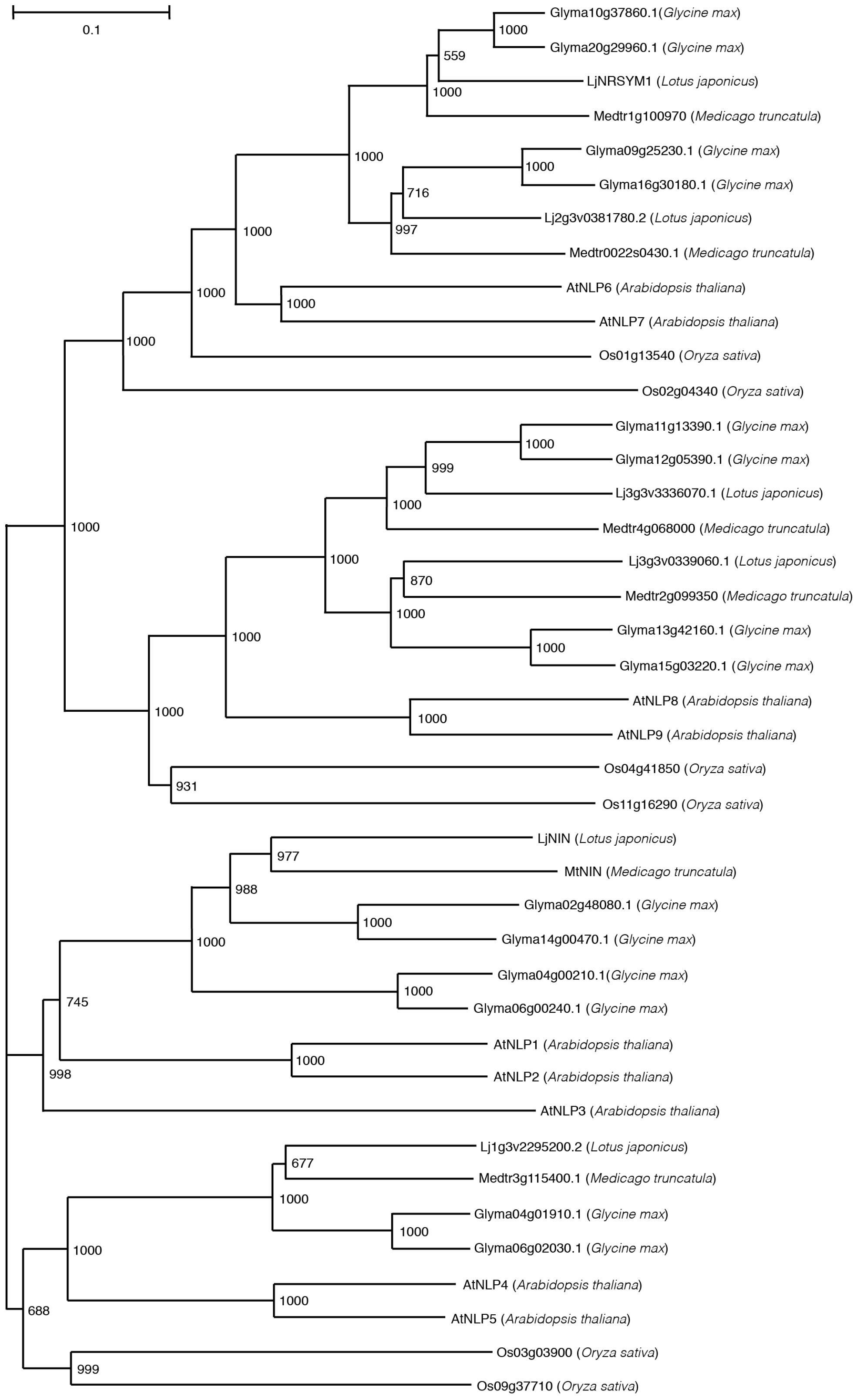

Supplementary Figure 3. A phylogenetic tree of the NLP family.

Full-length amino acid sequences were compared and the tree was constructed by the neighbor-joining method. The numbers are bootstrap values from 1000 replicates. Accession numbers of the amino acid sequences of related proteins are as follows: LjNRSYM1 (Lj5g3v1999250.2), LjNIN (Lj2g3v3373110.1), MtNIN (Medtr5g099060), AtNLP1 (AT2G17150.1), AtNLP2 (AT4G35270.1), AtNLP3 (AT4G38340.1), AtNLP4 (AT1G20640.1), AtNLP5 (AT1G76350.1), AtNLP6 (AT1G64530.1), AtNLP7 (AT4G24020.1), AtNLP8 (AT2G43500.1) and AtNLP9 (AT3G59580.1).

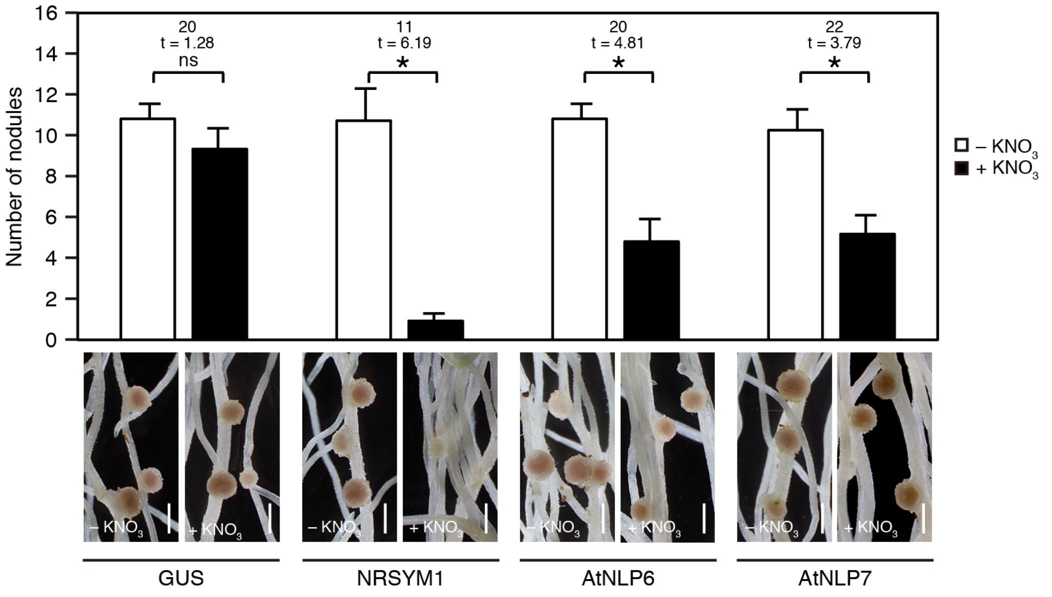

Supplementary Figure 4. Complementation of the *nrsym1* nodulation phenotype by the expression of *AtNLP6* or *AtNLP7*.

Nodulation and the number of nodules in transgenic hairy roots produced from the *nrsym1-1* mutants containing *pLjUBQ:GUS*, *pLjUBQ:NRSYM1*, *pLjUBQ:AtNLP6*, or *pLjUBQ:AtNLP7* constructs grown in the presence of 0 or 10 mM KNO_3 at 21 dai (n = 10–12 plants). Transgenic roots were identified by GFP fluorescence. *$P < 0.05$ by Student's *t*-test. ns, not significant. Degrees of freedom are shown above the *t*-values, Error bars indicate SEM. Scale bars: 1 mm.

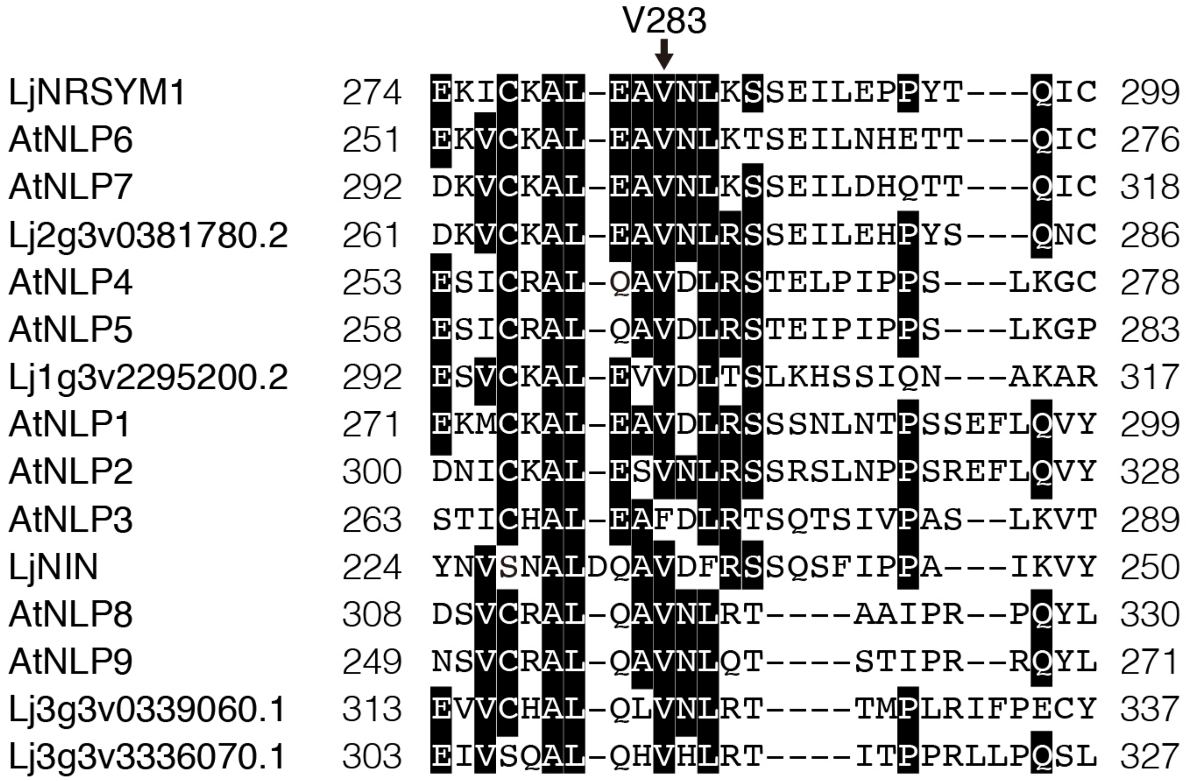

Supplementary Figure 5. Alignment of the amino acid sequences of *L. japonicus* and Arabidopsis NLPs.

Amino acid sequence alignment of a partial N-terminal conserved region containing the *nrsym1-2* mutation (V283) is shown. Amino acid residues that are conserved in more than half of the amino acid sequences are shaded in black. Accession numbers of the amino acid sequences of related proteins are as follows: LjNRSYM1 (Lj5g3v1999250.2), LjNIN (Lj2g3v3373110.1), AtNLP1 (AT2G17150.1), AtNLP2 (AT4G35270.1), AtNLP3 (AT4G38340.1), AtNLP4 (AT1G20640.1), AtNLP5 (AT1G76350.1), AtNLP6 (AT1G64530.1), AtNLP7 (AT4G24020.1), AtNLP8 (AT2G43500.1), and AtNLP9 (AT3G59580.1).

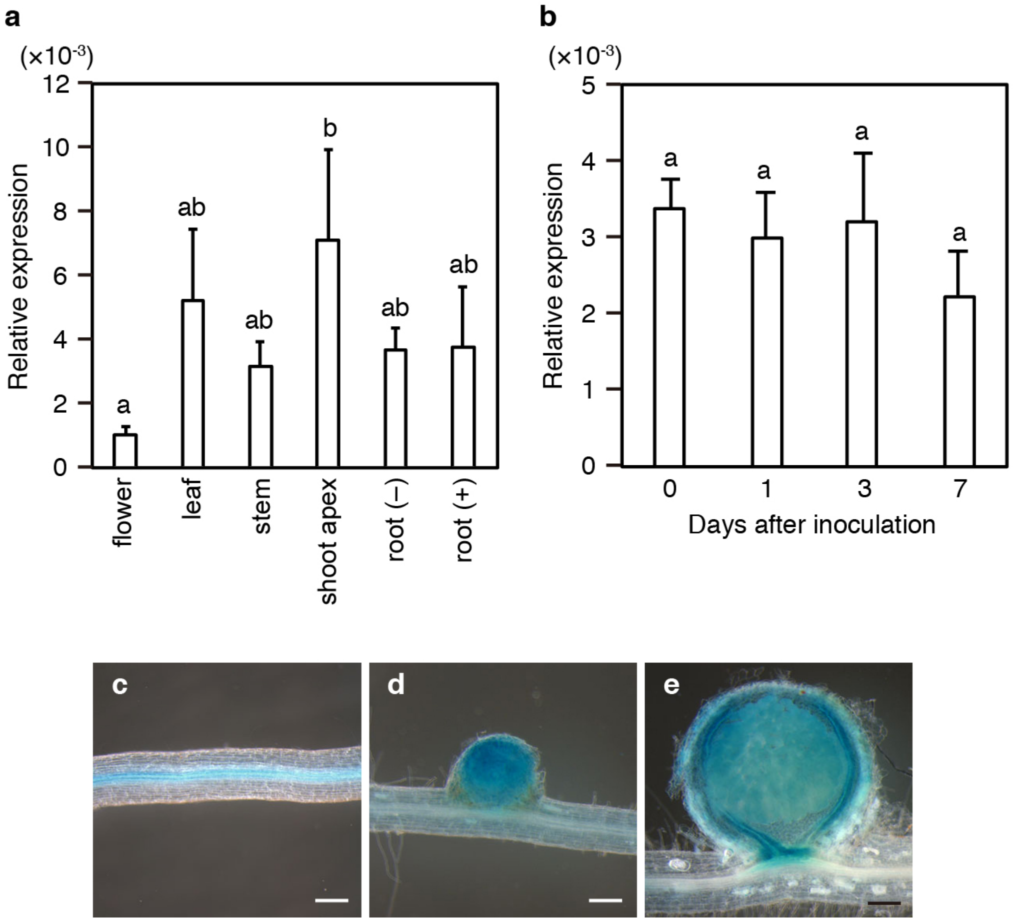

Supplementary Figure 6. The *NRSYM1* expression pattern.

(a) Real-time RT-PCR analysis of *NRSYM1* expression in WT. Each cDNA sample was prepared from total RNA derived from flowers, leaves, stems, shoot apices, or roots incubated with 0 (–) or 10 mM (+) KNO_3 for 24 h (n = 3 independent pools of roots). (b) Real-time RT-PCR analysis of *NRSYM1* expression in WT non-inoculated (0) and inoculated roots at 1, 3, and 7 dai under nitrate-free conditions (n = 4 independent pools of roots). Each cDNA sample was prepared from total RNA derived from a whorl of roots. The expression of *LjUBQ* was used as a reference. Error bars indicate SEM. Columns with the same lower-case letter indicate no significant difference (Tukey's test, $P < 0.05$). (c–e) Spatial expression patterns of *NRSYM1*. Blue staining indicates GUS activity under the control of the *NRSYM1* promoter in the (c) root, (d) nodule primordia and (e) a mature nodule of WT. GUS activity was observed at 10 dai (c,d) or 14 dai (e). Scale bars: 200 μm.

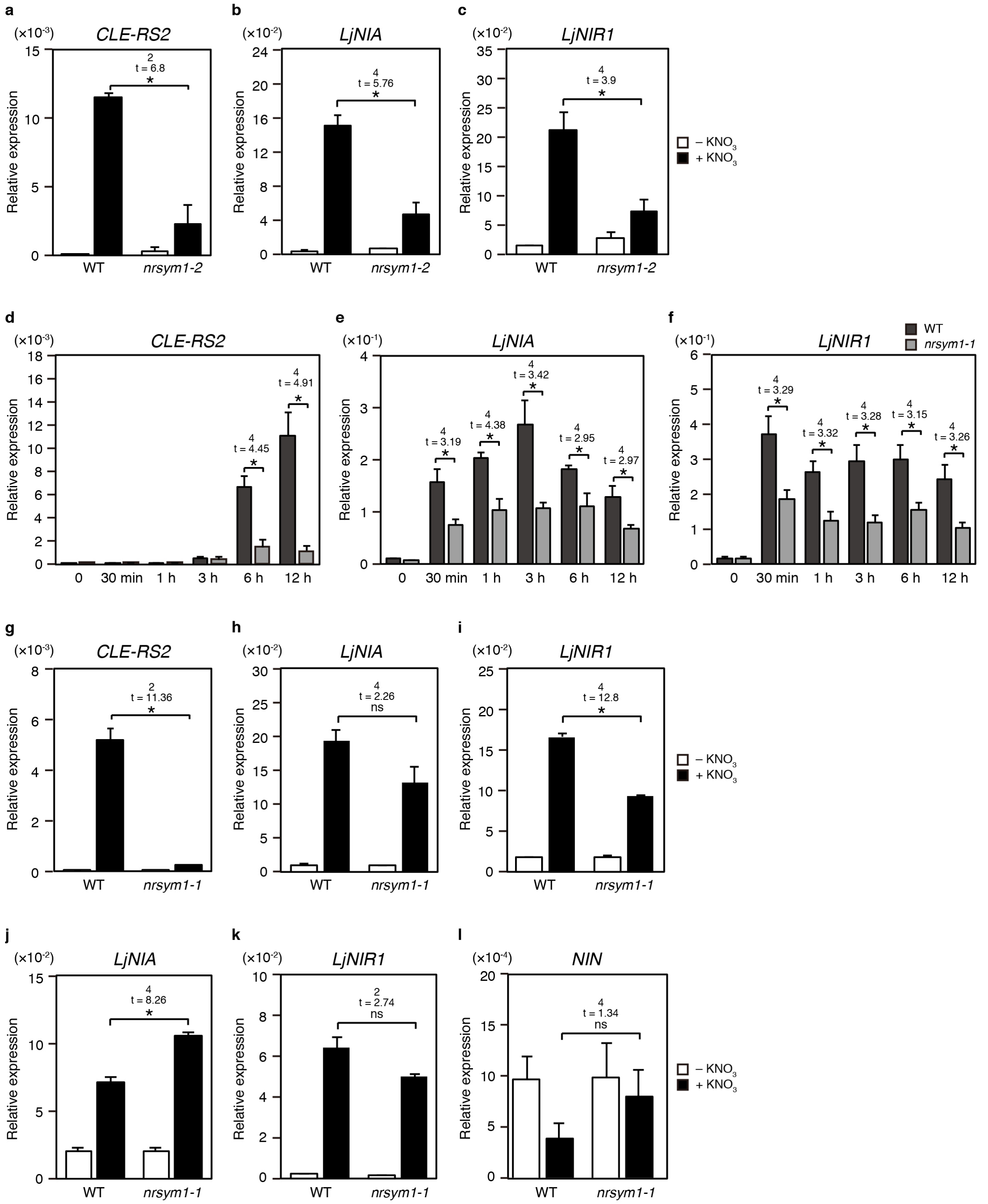

Supplementary Figure 7. The effect of the *nrsym1* mutation on gene expression.

(a–c) Real-time RT-PCR analysis of (a) *CLE-RS2*, (b) *LjNIA* and (c) *LjNIR1* expression in uninoculated roots of WT and the *nrsym1-2* mutants grown in the presence of 0 or 10 mM KNO_3 for 24 h. (d–i) Real-time RT-PCR analysis of (d,g) *CLE-RS2*, (e,h) *LjNIA,* and (f,i) *LjNIR1* in uninoculated roots of WT and the *nrsym1-1* mutants grown in N-free medium (0, $–KNO_3$) or in the presence of 10 mM KNO_3 for 30 min, 1, 3, 6, or 12 h (d–f) or 200 μM KNO_3 for 24 h (g–i). (j,k) Real-time RT-PCR analysis of (j) *LjNIA* and (k) *LjNIR1* in WT or *nrsym1-1* mutant leaves grown in the presence of 0 or 10 mM KNO_3 for 24 h. (l) Real-time RT-PCR analysis of *NIN* in 1 dai roots of WT and the *nrsym1-1* mutants grown in the presence of 0 or 10 mM KNO_3 for 24 h. Each cDNA sample was prepared from total RNA derived from (a–i,l) a whorl of roots or (j,k) leaves. The expression of *LjUBQ* was used as the reference. Error bars indicate SEM. (n = 3 independent pools of roots or leaves). *$P < 0.05$ by Student's *t*-test. ns, not significant. Degrees of freedom are shown above the *t*-values.

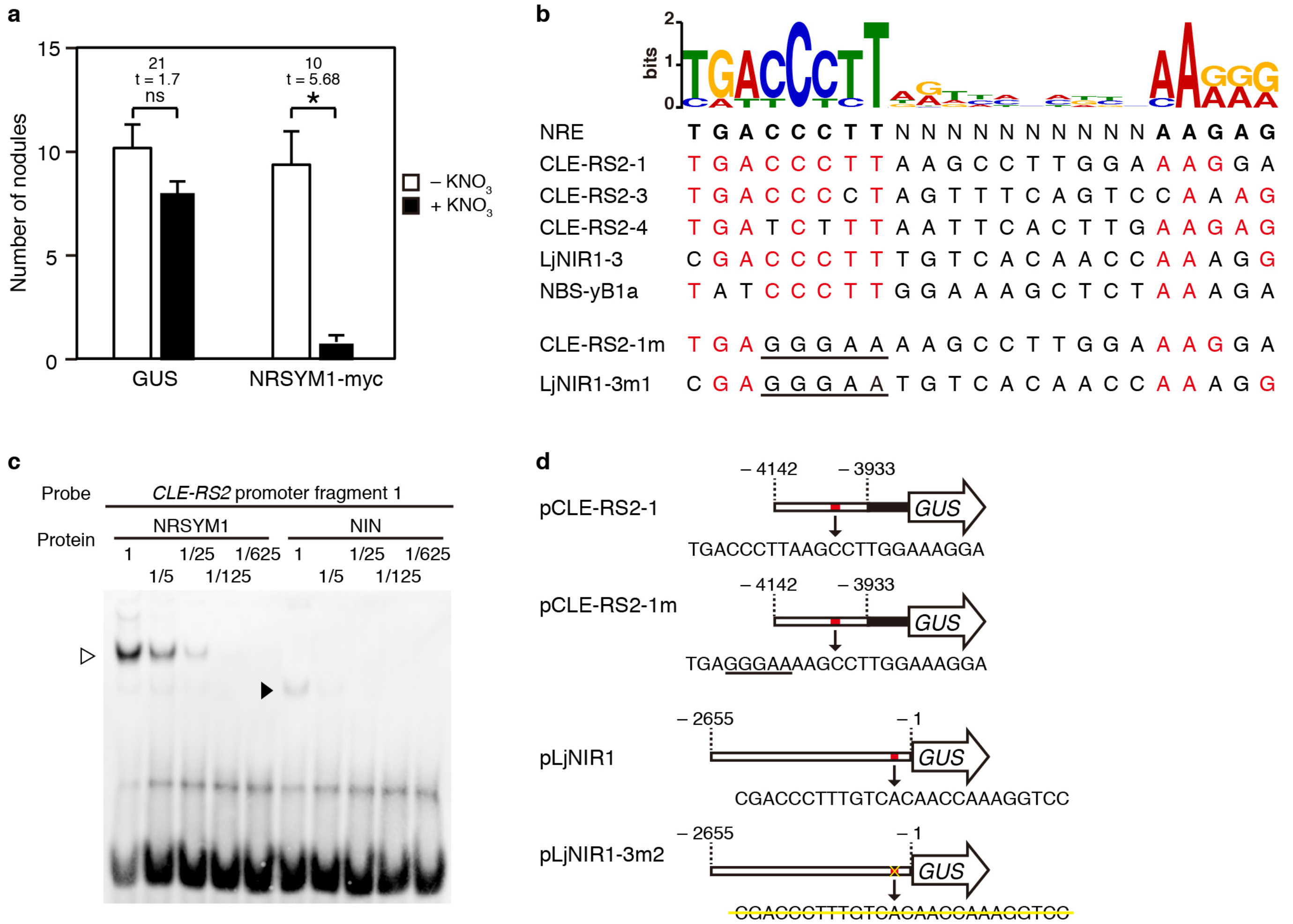

Supplementary Figure 8. Interaction of NRSYM1 and NIN with *CLE-RS2* promoters.

(a) The number of nodules in transgenic hairy roots produced from the *nrsym1-1* mutants containing either *pLjUBQ:GUS* or *pLjUBQ:NRSYM1-myc* constructs grown in the presence of 0 or 10 mM KNO_3 at 21 dai (n = 10–15 plants). Transgenic roots were identified by GFP fluorescence. *$P < 0.05$ by Student's *t*-test. ns, not significant. Error bars indicate SEM. Degrees of freedom are shown above the *t*-values. (b) Nucleotide sequences of the consensus sequence of NRE[1], NBS-yB1a[2] and NRE/NBS sequences that were identified in the *CLE-RS2* and *LjNIR1* promoter regions. Frequencies of nucleotide distributions among these sequences are shown with the MEME algorithm (http://meme-suite.org/index.html)[3]. Conserved nucleotide sequences among respective NRE/NBSs are shown in red letters. The mutated nucleotide sequences in CLE-RS2-1m and LjNIR1-3m1 are underlined. (c) EMSA showing NRSYM1 or NIN-binding with the *CLE-RS2* promoter 1. White and black arrowheads respectively indicate the position of shifted bands when the NRSYM1(531-976)-myc or NIN(520–878)-myc protein was incubated with the biotin-labeled probe. A dilution series of NRSYM1(531-976)-myc and NIN(520–878)-myc proteins were used for EMSA after adjusting for equal protein amounts by western blotting. (d) A schematic diagram of the promoter-*GUS* constructs used in Fig. 6f. Promoter fragments containing NRE/NBS (red bars) from the *CLE-RS2* and *LjNIR1* promoter regions were inserted upstream of the *GUS* gene. Black bars indicate the CaMV35S minimal promoter. The mutated nucleotide sequence in pCLE-RS2-1m is underlined. The entire NRE/NBS region is deleted in pLjNIR1-3m2.

a

CLE-RS1	1	5' – ATGATCTTCCAAACTACACCAATCTCTTCCATCATTCTACTAGCATCTTTGTTCTATTCTAAATCAGGCA – 3'	70
cle-rs1 #16	1	5' – ATGATCTTCCAAACTACACCAATCTCTTCCATCATTCTACTAGCATCTTTGTTCTATTCTAAATCAGGCA – 3'	70
CLE-RS1	71	5' – TGGAGAATGCAAGTGAAGTGCAAGTGTCGATGCTCATAGCAATGGTGTTCTGTACCTTGTTCGTGACTTT – 3'	140
cle-rs1 #16	71	5' – TGGAGAATGCAAGTGAAGTGCAAGTGTCGATGCTCATAGCAATGGTGTTCTGTACCTTGTTCGTGACTTT – 3'	140
CLE-RS1	141	5' – GCAGGCTCGTAGTCTCCATGAACAATATCCCTTGGTTCAGCAAAACATCAACAGCCTAGCCCTTCTGCAC – 3'	210
cle-rs1 #16	141	5' – GCAGGCTCGTAGTCTCCATGAACAATATCCCTTGGTTCAGCAAAACATCAACAGCCTAGCCCTTCTGCAC – 3'	210
CLE-RS1	211	5' – AAGTTAGGCATTGACCCATCAAAGCATGTACAGATTCGAGTTGATGATAGTAATGTCCCACTTTCACCAG – 3'	280
cle-rs1 #16	211	5' – AAGTTAGGCATTGACCCATCAAAGCATGTACAGATTCGAGTTGATGATAGTAATGTCCCACTTTCACCAG – 3'	280
CLE-RS1	281	5' – GAGATAGACTCTCACCA**GGAGGACCTGATCCTCAGCA**TAATGGAAAAAGACCACCCAGCAATCATCATTAG – 3'	351
cle-rs1 #16	281	5' – GAGATAGACTCTCACCA**GGA–GACCTGATCCTCAGCA**TAATGGAAAAAGACCACCCAGCAATCATCATTAG – 3'	351

CLE domain

CLE-RS2	1	5' – ATGGCGAAGACTACACTAGCTCGAGTAGTTTGTATATTTGTGCTAGTTATCATCTTCTCC – 3'	60
cle-rs2 #2	1	5' – ATGGCGAAGACTACACTAGCTCGAGTAGTTTGTATATTTGTGCTAGTTATCATCTTCTCC – 3'	60
cle-rs2 #5	1	5' – ATGGCGAAGACTACACTAGCTCGAGTAGTTTGTATATTTGTGCTAGTTATCATCTTCTCC – 3'	60
CLE-RS2	61	5' – AACTTCTTCATGACATTGCAGGCTCGTAATCTCCAAATCATTCACAAAAACAATGCAGTT – 3'	120
cle-rs2 #2	61	5' – AACTTCTTCATGACATTGCAGGCTCGTAATCTCCAAATCATTCACAAAAACAATGCAGTT – 3'	120
cle-rs2 #5	61	5' – AACTTCTTCATGACATTGCAGGCTCGTAATCTCCAAATCATTCACAAAAACAATGCAGTT – 3'	120
CLE-RS2	121	5' – CAAAATTATGTTTTTGACCTATCAAAGCACATGCACGTTGTTCACAAGGATGGATATCAA – 3'	180
cle-rs2 #2	121	5' – CAAAATTATGTTTTTGACCTATCAAAGCACATGCACGTTG-------------------- – 3'	180
cle-rs2 #5	121	5' – CAAAATTATGTTTTTGACCTATCAAAGCACATGCACGTTGTTCACAAGGATGGATATCAA – 3'	180
CLE-RS2	181	5' – CAGCAAAGACTCTCACCT**GGAGGACCAGATCCTCAACA**TAATAATGCAATACCTCCAAGC – 3'	240
cle-rs2 #2	181	5' – ----------------------------- **TCCTCAACA**TAATAATGCAATACCTCCAAGC – 3'	240
cle-rs2 #5	181	5' – CAGCAAAGACTCTCACCT**GGAGGGACCAGATCCTCAACA**TAATAATGCAATACCTCCAAGC – 3'	240

CLE domain

CLE-RS2	241	5' – AATTAG – 3'	246
cle-rs2 #2	241	5' – AATTAG – 3'	246
cle-rs2 #5	241	5' – AATTAG – 3'	246

b

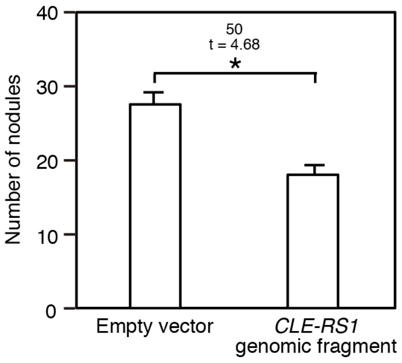

Supplementary Figure 9. The position of mutations in *cle-rs1* and *-rs2* plants created by the CRISPR/Cas9 genome editing system and complementation of the *cle-rs1 -rs2* double mutant nodulation phenotype.

(a) Nucleotide alignment of *CLE-RS1* and *-RS2* genes. The indel mutations near the protospacer adjacent motif (PAM) site (blue letters) of *CLE-RS1* and *-RS2* are shown in red. The sgRNA target is indicated in bold letters. The nucleotides sequences that encode amino acids of the CLE domains are underlined. (b) The number of nodules in transgenic hairy roots produced from the *cle-rs1 -rs2* double mutants carrying either a control empty vector or a 7.4-kb genomic fragment encompassing the entire *CLE-RS1* locus at 21 dai (n = 26 plants). Transgenic roots were identified by GFP fluorescence. *$P < 0.05$ by Student's t-test. Degrees of freedom are shown above the t-values. Error bars indicate SEM.

Supplementary Table 1. SNP filtering in the *nrsym1* mutants

	**WT (MG20)**	***nrsym1-1***	***nrsym1-2***
Total raw reads	125,479,432	178,108,716	102,628,550
Total nucleotides (Mb)	12,673	17,988	10,365
Coverage	27.0x	38.3x	22.1x
Total SNP / genome	378,841	403,934	366,454
Number of homo-type SNP candidates / genome	85,519	93,569	82,360
Comparison of SNP candidates of MG20 / genome	-	22,509	17,167
C to T, G to A / genome	-	4,320	3,541
SNPs in the exon and intron acceptor and donor site / genome	-	350	304
F2 plants used for rough mapping	-	15	15
Mapped region	-	6.0 Mb	6.0 Mb
Gene number annotated / mapped region	-	561	561
SNPs in the exon and intron acceptor/donor site / mapped region	-	3	1
Shared SNPs in same genes	-	chr5.CM0148.170.r2.a	

Supplementary Table 2. EMSA probes

EMSA-RS2-1-F	5'–ggtgTGTGAGTTCTGACCCTTAAGCCTTGGAAAGGACAGTCATGCAA–3'
EMSA-RS2-1-R	5'–ggtgTTGCATGACTGTCCTTTCCAAGGCTTAAGGGTCAGAACTCACA–3'
EMSA-RS2-2-F	5'–ggtgGGTTTACGACCTAACTCATTGAGGCATGATGTGACTTGACTTGT–3'
EMSA-RS2-2-R	5'–ggtgACAAGTCAAGTCACATCATGCCTCAATGAGTTAGGTCGTAAACC–3'
EMSA-RS2-3-F	5'–ggtgGTATCAAACTGACCCCTAGTTTCAGTCCAAAGCCACCTTGATTG–3'
EMSA-RS2-3-R	5'–ggtgCAATCAAGGTGGCTTTGGACTGAAACTAGGGGTCAGTTTGATAC–3'
EMSA-RS2-4-F	5'–ggtgATATATTCATGATCTTTAATTCACTTGAAGAGTTAGTGATTGAT–3'
EMSA-RS2-4-R	5'–ggtgATCAATCACTAACTCTTCAAGTGAATTAAAGATCATGAATATAT–3'
EMSA-RS2-1m-F	5'–ggtgTGTGAGTTCTGAGGGAAAAGCCTTGGAAAGGACAGTCATGCAA–3'
EMSA-RS2-1m-R	5'–ggtgTTGCATGACTGTCCTTTCCAAGGCTTTTCCCTCAGAACTCACA–3'
EMSA-LjNIR1-2-F	5'–ggtgTCATGTACCCCCCTTCCCAAAGTAGGAAGAGGTCGTCCCTCAAC–3'
EMSA-LjNIR1-2-R	5'–ggtgGTTGAGGGACGACCTCTTCCTACTTTGGGAAGGGGGGTACATGA–3'
EMSA-LjNIR1-3-F	5'–ggtgACACAAACACGACCCTTTGTCACAACCAAAGGTCCATTGTAGCA–3'
EMSA-LjNIR1-3-R	5'–ggtgTGCTACAATGGACCTTTGGTTGTGACAAAGGGTCGTGTTTGTGT–3'
EMSA-LjNIR1-3m1-F	5'–ggtgACACAAACACGAGGGAATGTCACAACCAAAGGTCCATTGTAGCA–3'
EMSA-LjNIR1-3m1-R	5'–ggtgTGCTACAATGGACCTTTGGTTGTGACATTCCCTCGTGTTTGTGT–3'

Supplementary Table 3. Primers used in this work

Map-based cloning of *NRSYM1*			
	TM0913-F	5'–ATGAAGGTACTGCATTCCAC–3'	
	TM0913-R	5'–TTTGCCATGGTTCAATTCTG–3'	
	TM1417-F	5'–GGCTTTTGAAAGAGATCCAG–3'	
	TM1417-R	5'–GGAGGTATATTTAGTGCAGGG–3'	
	TM0366-F	5'–CAGGGGATTTTATCATGGGG–3'	
	TM0366-R	5'–CATCCGGGTCTCTGACCCTC–3'	
	TM0698-F	5'–CGCATCCATCACCTCTTTTC–3'	
	TM0698-R	5'–GTGACCTGTTCACAGTTTCG–3'	
Sequencing of *NRSYM1*			
	NRSYM1-F1	5'–TGAGTTGCCTTGAAGGTTTG–3'	
	NRSYM1-R1	5'–GGTAGTTGATGCCACAACAAGA–3'	
	NRSYM1-F2	5'–AAGTGTATCCACAGGTCGCT–3'	
	NRSYM1-R2	5'–TGAAGTTCATCCTGTGGTTA–3'	
	NRSYM1-F3	5'–GTATTTCCAGCTAGCAGAAG–3'	
	NRSYM1-F4	5'–CACATAATCATCATCTCCTG–3'	
	NRSYM1-F5	5'–GGTACACATCAAACATAAGC–3'	
Complementation of *nrsym1*			
	NRSYM1-F6	5'–CAAGTCGACCATACGCACCGCTCAGAAATCG–3'	SalI site is added
	NRSYM1-R3	5'–ACCGTCGACGGAGTGTTAGGTTGCTACCTGG–3'	SalI site is added
Cloning of *NRSYM1* cds			
	NRSYM1-F8	5'–CACCATGTCAGAATCTGATGAAGA–3'	
	NRSYM1-R5	5'–TCACTCCCCTGAGCTCTCAC–3'	
Cloning of *AtNLP6* cds			
	NLP6-F1	5'–CACCATGGAACTTGACGACTTGGA–3'	
	NLP6-R1	5'–TCACAAGCACATCATAGTTT–3'	
Cloning of *AtNLP7* cds			
	NLP7-F1	5'–CACCATGTGCGAGCCCGATGATAA–3'	
	NLP7-R1	5'–TCACAATTCTCCAGTGCTCT–3'	
NRSYM1 RT-PCR			
	NRSYM1-RT-PCR-F	5'–GCATCACTTACAACAAGGTCAAGG–3'	
	NRSYM1-RT-PCR-R	5'–TGGGTAATGTTTGGGCAGAAG–3'	
Cloning of *NRSYM1* promoter			
	NRSYM1-F7	5'–CAAGAGCTCGGAGTGTTAGGTTGCTACCTGG–3'	SacI site is added
	NRSYM1-R4	5'–CAAGGTACCTTTTCTCTCTCAGGGTGCTAAC–3'	KpnI site is added
LjUBQ RT-PCR			
	UBQ-RT-PCR-F	5'–ATGCAGATCTTCGTCAAGACCTTG–3'	
	UBQRT-PCR-R	5'–ACCTCCCCTCAGACGAAG–3'	
CLE-RS1 RT-PCR			
	RS1-RT-PCR-F	5'–TGCAAGTGTCGATGCTCATAGC–3'	
	RS1RT-PCR-R	5'–GATGTTTTGCTGAACCAAGGGATA–3'	
CLE-RS2 RT-PCR			
	RS2-RT-PCR-F	5'–GCTCGTAATCTCCAAATCATTCACA–3'	
	RS2-RT-PCR-R	5'–GGTGAGAGTCTTTGCTGTTGATATCC–3'	

Supplementary Table 3. (continued)

LjNIA RT-PCR			
	LjNIA1-RT-PCR-F	5'–GAAGGACCCAGAGGATCACA–3'	
	LjNIA1-RT-PCR-R	5'–CGGTCTTCGTACTTCTTCGC–3'	
LjNIR1 RT-PCR			
	LjNIR1-RT-PCR-F	5'–GCAAGTGCAGGTTGCTGATA–3'	
	LjNIR1-RT-PCR-R	5'–CTTCCTATCCTCCCTCCCAG–3'	
LjNIN RT-PCR			
	LjNIN-RT-PCR-F	5'–CAATGCTCTTGATCAGGCTGTTGA–3'	
	LjNIN-RT-PCR-R	5'–GAGTGCTAATGGCAAATTGTGTGTC–3'	
Cloning of *NRSYM1* cds without a stop codon			
	NRSYM1-F8	5'–CACCATGTCAGAATCTGATGAAGA–3'	
	NRSYM1-R6	5'–CTCCCCTGAGCTCTCACATG–3'	
Cloning of *NRSYM1-myc*			
	NRSYM1-F8	5'–CACCATGTCAGAATCTGATGAAGA–3'	
	NRSYM1-R7	5'–TGAACGATCGGGGAAATTCG–3'	
CLE-RS2 ChIP-qPCR			
	ChIP-RS2-1-F	5'–CTTCATATCAATCTTGAGGCTG–3'	
	ChIP-RS2-1-R	5'–GGCTTAAGAAAACTCTTTGGC–3'	
	ChIP-RS2-2-F	5'–GCTACGAGGCAGCTGTTAGG–3'	
	ChIP-RS2-2-R	5'–TAATATTACATGAACAAGTTAGTTTATCAAGCA–3'	
	ChIP-RS2-3-F	5'–AGACCTTATCCTATCAAGCCTAATG–3'	
	ChIP-RS2-3-R	5'–CTAACGTATGTATGTGGACAAATAGG–3'	
	ChIP-RS2-4-F	5'–CACCTTGATTGGGCAGTACTTC–3'	
	ChIP-RS2-4-R	5'–GTTAGAAGGATCCGAAGTGAAAATG–3'	
LjNIR1 ChIP-qPCR			
	ChIP-LjNIR1-1-F	5'–CCCTAAAATCGGTCATAAACCC–3'	
	ChIP-LjNIR1-1-R	5'–GGTTTAGGGTTTAGGGATAGGG–3'	
	ChIP-LjNIR1-2-F	5'–TTGTGCTCTCATCCACCTCA–3'	
	ChIP-LjNIR1-2-R	5'–CGGTTTGGCTAAGGATGCTA–3'	
	ChIPLjNIR-1-3-F	5'–CCTCCATTTTGACTAACCATGTGC–3'	
	ChIP-LjNIR1-3-R	5'–GGAGTGGAACCTTCCGTGAAG–3'	
Cloning of *NRSYM1-myc* for EMSA			
	NRSYM1-F9	5'–ATTGTCGACATGGAGGAAGTGCCAAAGGATC–3'	SalI site is added
	NRSYM1-R8	5'–ATTGCGGCCGCTCACTCCCCTGAGCTCTCAC–3'	NotI site is added
	Myc-F1	5'–ATTGCGATCGCGGAACCAATTCAGTCGAGAT–3'	SgfI site is added
	NRSYM1-R9	5'–ATTGTTTAAACTCACTCCCCTGAGCTCTCAC–3'	PmeI site is added
GUS RT-PCR			
	GUS-RT-PCR-F	5'–TAACGATCAGTTCGCCGATG–3'	
	GUS-RT-PCR-R	5'–TTTGCCGTAATGAGTGACCG–3'	
GFP RT-PCR			
	GFP-RT-PCR-F	5'–TATATCATGGCCGACAAGCA–3'	
	GFP-RT-PCR-R	5'–TGTTCTGCTGGTAGTGGTCG–3'	

Supplementary Table 3. (continued)

Cloning of *LjNIR1* promoter			
	LjNIR1-F1	5'–ATT<u>GAGCTC</u>GCCACATATGCGATGACTGG–3'	SacI site is added
	LjNIR1-R1	5'–ATT<u>CCCGGG</u>GGTGAGGTGAGGGAGTGTGG–3'	SmaI site is added
	LjNIR1-F2	5'–ATT<u>GGTACC</u>ATTGTAGCAACAAAGAACCTC–3'	KpnI site is added
	LjNIR1-R2	5'–ATT<u>GGTACC</u>TGTTTGTGTTTGATTCAACTGG–3'	KpnI site is added
sgRNA of CLE-RS1			
	RS1-F1	5'–ATTGTGCTGAGGATCAGGTCCTCC–3'	
	RS1-R1	5'–AAACGGAGGACCTGATCCTCAGCA–3'	
sgRNA of CLE-RS2			
	RS2-F1	5'–ATTGTGTTGAGGATCTGGTCCTCC–3'	
	RS2-R1	5'–AAACGGAGGACCAGATCCTCAACA–3'	
Complementation of *cle-rs1*			
	RS1-F2	5'–ATT<u>GGTACC</u>GTCTTCCTAGAAGTGGGCTAGG–3'	KpnI site is added
	RS1-R2	5'–ATT<u>GGATCC</u>TGACATTAGGGCGTCGCAGTAG–3'	BamHI site is added
Sequencing of *CLE-RS1*			
	RS1-RT-PCR-F	5'–TGCAAGTGTCGATGCTCATAGC–3'	
	RS1-R3	5'–AAAGAGGAGTGCAGACGGAA–3'	
Sequencing of *CLE-RS2*			
	RS2-F2	5'–GCAGGCTCGTAATCTCCAAA–3'	
	RS2-R2	5'–GGCTCTTCATTGCTTTCCAG–3'	
Sequencing of *LjCLE49*			
	CLE49-F1	5'–CCTCTGAACCCAACAGTGGT–3'	
	CLE49-R1	5'–CCCACCATGCTGTGATTTTA–3'	
Sequencing of *CLE-RS3*			
	RS3-F1	5'–GGGGTTCCACCTTATGGAGT–3'	
	RS3-R1	5'–TCACACCGCAGAGAAGAGAA–3'	

Supplementary References

1 Konishi, M. & Yanagisawa, S. Roles of the transcriptional regulation mediated by the nitrate-responsive *cis*-element in higher plants. *Biochem. Biophys. Res. Commun.* **411,** 708-713 (2011).

2 Soyano, T., Kouchi, H., Hirota, A. & Hayashi, M. NODULE INCEPTION directly targets *NF-Y* subunit genes to regulate essential processes of root nodule development in *Lotus japonicus*. *PLOS Genet.* **9,** e1003352 (2013).

3 Bailey, T. L. *et al.* MEME Suite: tools for motif discovery and searching. *Nucleic Acids Res.* **37,** W202-W208 (2009).
